# The familial experience of acute bacterial meningitis in children. A transversal qualitative study using interpretative phenomenological analysis

**DOI:** 10.1192/j.eurpsy.2021.1058

**Published:** 2021-08-13

**Authors:** E. Scanferla, P. Gorwood, L. Fasse

**Affiliations:** 1 Ed 450, Université de Paris, Paris, France; 2 Ea 4403, Clipsyd, Université Paris Nanterre, France, France; 3 Cmme, GHU Paris psychiatrie neurosciences, Paris, France; 4 Institute Of Psychiatry And Neuroscience Of Paris, INSERM, Paris, France; 5 Lpps, Ea 4057, Université de Paris, Boulogne Billancourt, France; 6 Département De Soins De Support, Gustave Roussy Institute, Villejuif, France

**Keywords:** Subjective experience, Meningitis, survivors, Meaning-making process, Qualitative methods

## Abstract

**Introduction:**

Pediatric acute bacterial meningitis is a life-threatening illness that results from bacterial infection of the meninges and leaves some survivors with significant sequelae. Given the potential trauma induced by the disease itself and the hospitalization, it is important to have an insight on how the parents cope with this aversive event, and especially how they give sense to this experience.

**Objectives:**

(1) To explore the lived experience of close family ascendants whose child or grandchild had survived acute bacterial meningitis (2) To investigate how they give meaning to this specific experience.

**Methods:**

Participants were recruited through two association of persons affected by meningitis. Convenience sample of eleven family ascendants. Their family descendants were aged between 0.2 and 20 years old at the time of the meningitis diagnosis (M= 4.1, SD= 7.3). In average, 9.4 years had passed between the onset of illness and the relative’s interview (SD= 5.4).

**Results:**

6 superordinate themes and 2 meaning-making processes were identified: 1. Sick child becoming a “hero” (comparison with other children). 2. Engaged action/attitude: finding the “positive” of the traumatic experience and engaged action to improve the care system.

**Conclusions:**

This is one of the first studies exploring the first-hand experience of family ascendants confronted to acute bacterial meningitis. Findings highlighted factors characterising the disease experience and the psychological adjustment of meningitis survivors’ families. They demonstred (1) the multidimensional impact of the disease on family ascendants and their need for professional psychological support, (2) the importance of direct involvement of parents in identifying key aspects of care.

